# Focal adhesion kinase (FAK) expression and activation during lens development

**Published:** 2007-03-26

**Authors:** Maria I. Kokkinos, Heidi J. Brown, Robbert U. de Iongh

**Affiliations:** 1 Department of Anatomy & Cell Biology, University of Melbourne, Parkville, Australia; 2 Department of Anatomy & Histology and Institute for Biomedical Research, University of Sydney, Sydney, Australia

## Abstract

**Purpose:**

Regulation of lens development involves an intricate interplay between growth factor (e.g. FGF and TGFβ) and extracellular matrix (ECM) signaling pathways. Focal adhesion kinase (FAK) is a cytoplasmic tyrosine kinase that plays key roles in transmitting ECM signals by integrins. In this study, we delineated patterns of FAK expression and tyrosine phosphorylation (Y^397^) in the developing lens and investigated its regulation by FGF2. We also examined FAK expression and activation during disrupted fiber differentiation in mice expressing a dominant-negative TGFβ receptor.

**Methods:**

FAK expression and activation (phosphorylation on Y^397^) was studied in embryonic and postnatal rodent lenses by in situ hybridization, immunofluorescence, and western blotting. Rat lens explants were used to investigate the effects of FGF2 on FAK expression and activation. Immunofluorescence and western blotting were used to examine FAK expression and phosphorylation in transgenic mice that express a dominant-negative TGFβ receptor.

**Results:**

FAK is widely expressed and phosphorylated during embryonic stages of lens morphogenesis and differentiation. However, in postnatal lenses its expression and activation becomes restricted to the posterior germinative zone and the transitional zone at the lens equator. While both NH_2_- and COOH-terminal antibodies revealed cytoplasmic and membrane-associated staining in lens cells, the NH_2_-terminal antibody also showed FAK was present in fiber cell nuclei. In vitro, FAK expression and phosphorylation on Y^397^ were increased by concentrations of FGF2 that initiate lens epithelial cell migration (10 ng/ml) and differentiation (50 ng/ml) but not proliferation (5 ng/ml). Moreover, reactivity for Y^397^phosphorylated FAK is prominent in the nuclei of differentiating fibers both in vivo and in vitro. Disruption of TGFβ-like signals by ectopic expression of a dominant-negative TGFβ receptor (TβRII^D/N^) results in abnormal lens fiber differentiation in transgenic mice. While FAK expression is initiated normally in the posterior germinative zone of TβRII^D/N^ transgenic lenses, as fiber differentiation proceeds, FAK becomes localized to a perinuclear compartment, decreases its association with the cytoskeleton and is poorly phosphorylated on Y^397^.

**Conclusions:**

FAK is widely expressed and activated during early lens morphogenesis. During secondary lens fiber differentiation, FAK is expressed and phosphorylated on Y^397^ as epithelial cells exit the cell cycle, initiate migration at the equator, and undergo differentiation in the transitional zone. During terminal fiber differentiation an NH_2_-terminal fragment of FAK, including Y^397^, is translocated to the nucleus. The expression, activation, and nuclear localization of FAK are regulated, at least partly, by FGF2. FAK activity and subcellular localization are also modulated by TGFβ-like signals. In fiber cells of TβRII^D/N^ transgenic lenses, FAK is abnormally retained in a perinuclear compartment, loses its association with the cytoskeleton, and is poorly phosphorylated. These data suggest that integrin signaling via FAK plays important roles during lens differentiation, mediated by FGFs and TGFβ-superfamily signals.

## Introduction

Lens development involves an intricate network of regulatory genes and interplay between growth factor and extracellular matrix (ECM) signaling pathways. Lens morphogenesis is initiated by a series of inductive interactions, culminating in contact between the neuroepithelium of the presumptive retina (optic vesicles) and the presumptive lens ectoderm [1]. Upon contact with the optic vesicles, the lens ectoderm thickens to form a placode that subsequently invaginates to form a vesicle. The anterior vesicle cells proliferate and form the lens epithelium, whereas the posterior cells (facing the optic cup) undergo extensive elongation and differentiation to form primary lens fibers [2-4]. During fetal and postnatal development, cell proliferation becomes increasingly localized to a band of epithelial cells above the lens equator, the germinative zone [5]. As the lens grows, progeny of these divisions move below the equator, into the transitional zone, where they elongate and differentiate into secondary lens fibers [2]. Terminal fiber differentiation is characterized by the abrupt loss of nuclei and organelles from the cortical fibers by an apoptotic or autophagic mechanism [6-9].

Several growth factor signaling pathways have been implicated in regulating lens development [10]. In particular, FGFs have been shown to regulate the patterns of cell proliferation, migration, and differentiation in the lens, leading to its highly polarized structure. FGFs are the principal inducers of fiber differentiation, but other factors, notably members of the TGFβ superfamily, have been shown to be required for fiber elongation and terminal differentiation [11-15]. More recently, the family of Wnt growth factors has been implicated at various stages of lens induction and differentiation [16-18].

During lens morphogenesis, the ectodermal basement membrane that surrounds the lens vesicle thickens to form the lens capsule. It is composed of laminin, heparan sulfate proteoglycans (HSPG), SPARC, entactin/nidogen, fibronectin, and collagen IV [19-22]. The lens capsule has been shown to modulate, and to be modulated by, growth factors. For example, the capsule sequesters FGFs [23-26] and different regions of the capsule have different proliferative effects on lens epithelial cells due to different presentation of FGF [27]. Conversely, inappropriate stimulation of the lens epithelium by TGFβ results in dramatic changes in the expression of ECM proteins such as laminins, fibronectin, HSPG, and collagens I and III, which accompany the epithelial to mesenchymal transition, characteristic of subcapsular cataract [28]. Similarly, alteration in composition of the lens capsule, such as occurs in the SPARC-null mutant mouse, results in dramatic changes in lens transparency and gene expression [29,30], indicating that the ECM of the lens capsule is an important regulator of cellular processes in the lens.

Integrins are cell surface receptors for ECM proteins, such as laminin, fibronectin, and collagen, which mediate cell adhesion and modulate growth factor signaling. Integrins are composed of non-covalently associated, heterodimeric transmembrane polypeptides (α- and β-subunits) that are involved in bidirectional signaling ("inside-out" and "outside-in") [31]. Intracellular signals, often as a result of growth factor signals, affect integrin activity on the surface by initiating conformational changes that expose ligand binding sites. ECM ligand binding subsequently leads to the assembly of multiprotein complexes in association with the integrin cytoplasmic tails. These complexes link to the actin cytoskeleton and include kinases that modulate many intracellular signaling pathways [31-33].

Recent studies have shown that the lens expresses the laminin binding integrins α3β1, α6Aβ1, and α6Bβ1 in patterns suggestive of distinct roles in lens development [34-36]. Moreover, the integrins are differentially regulated, with α6 splice variant expression being regulated by FGF and α3 expression being regulated by an unidentified factor in vitreous [36]. Integrins are required at various stages of lens development (reviewed in [37]). Injection of antibodies or RGD peptides into the preoptic regions of chick embryos, to block integrin function, results in a failure of eye morphogenesis [38]. Double null mutation of α3 and α6 integrin genes results in abnormalities of the anterior lens epithelium during embryonic development [39]. However, how integrin signaling affects lens cell processes is not well understood.

Focal adhesion kinase (FAK) is a protein tyrosine kinase involved in "outside-in" integrin signaling and plays important roles during cell migration and proliferation in both normal and transformed cells [40,41]. FAK becomes rapidly phosphorylated by integrin engagement of ECM ligands or by growth factor signals. Activation of FAK at focal adhesions promotes cell motility, enhances cell survival, increases cell proliferation via the MAPK pathway, and is associated with differentiation of several cell types [42,43]. FAK consists of large NH_2_- and COOH-terminal domains, which flank an internal catalytic region. The NH_2_-terminal FERM (protein 4.1. ezrin, radixin, moesin homology) domain interacts with integrins and receptor tyrosine kinases. The COOH-terminal domain contains the FAT (focal adhesion targeting) domain that acts as a binding site for focal adhesion proteins (particularly paxillin and talin) and two proline-rich regions that act as binding sites for SH_3_ domain-containing proteins. The COOH-terminal noncatalytic region is also expressed independently, from an alternative intron-located promoter, as a variant of FAK called FRNK (FAK-related non-kinase) and is thought to act as a dominant-negative inhibitor. FAK contains six known sites of tyrosine phosphorylation, which are associated with various activities of FAK. The major and best characterized phosphorylation site is tyrosine-397 (Y^397^), located just upstream of the kinase domain. Phosphorylated Y^397^ is a binding site for a number of SH_2_ domain-containing proteins, including the Src family kinases [40,41] and binding triggers Src activation. Activated Src can then induce further phosphorylation of other FAK tyrosine residues, resulting in maximal activation of FAK and generation of binding sites for various other adaptor proteins such as Grb2, which can activate the MAPK pathway. Other proteins with SH_2_ sites that can bind to Y^397^ include PI3 kinase, PLCγ, and Grb7. This autophosphorylation promotes the initiation of FAK-related signaling complexes at focal adhesion sites. Conversely, displacing FAK from focal adhesions inhibits its tyrosine phosphorylation, impairing its function [40,41].

In this study, we delineate the expression and activation of FAK in the developing lens and investigate its regulation by FGF. We also examine whether FAK expression or activation is altered in lenses of mice with disrupted terminal fiber differentiation due to ectopic expression of a dominant-negative TGFβ receptor. The results suggest that FAK plays important roles during lens differentiation, particularly as cells exit the cell cycle in the germinative zone and initiate fiber differentiation in the transitional zone. During lens fiber differentiation, FAK expression and phosphorylation are modulated by FGF2 and phosphorylated FAK or a FAK NH_2_-terminal fragment is targeted to the nucleus. In fibers undergoing abnormal terminal differentiation due to expression of TβRII^D/N^, FAK is abnormally targeted within the cell, becomes disassociated from the cytoskeleton and is poorly phosphorylated.

## Methods

### Tissues

All animal procedures were performed in accordance with the Association for Research and Ophthalmology (ARVO) Statement for the Use of Animals in Ophthalmic and Vision Research and were approved by the Animal Ethics Committee of the University of Melbourne.

Ocular embryonic (E) and post-natal (P) tissues were obtained from Wistar rats and from wild-type (FVB/N) and TβRII^D/N^ transgenic (OVE591) mice. The transgenic mice (OVE591) over-expressing the dominant negative truncated TGFβ receptor II (TβRII^D/N^) have been described previously [12]. Transgenic mice were identified by PCR of SV40 transgene sequences from genomic DNA as described previously [12]. Embryonic (E) tissues were collected from super-ovulated (5 IU Folligon; 5 IU Chorulon, Intervet, Sydney, NSW Australia) pregnant females euthanized on days E11.5, E13.5, E14.5, and E17.5. Postnatal eyes were obtained from euthanized neonatal (P1-3) and weanling (P21) animals. Tissues were used for tissue culture, protein extraction or were fixed overnight in 10% neutral buffered formalin (NBF), transferred to 70% ethanol and embedded in paraffin.

### Lens epithelial explants

Lens epithelial explants were prepared from P9-14 Sprague Dawley or Wistar rats and cultured in Medium 199 (Thermo-Trace, Noble Park, VIC, Australia) with or without recombinant human FGF2 (0-50 ng/ml; PeproTech, Rocky Hill, NJ) as described previously [36] for up to 8 days. Medium and growth factors were replenished every three days. Following incubation, explants were collected either for immunofluorescence or for western blotting.

### In situ hybridization

To generate in situ hybridization probes, cDNAs within the coding domain of murine FAK (Genbank M95408) were amplified by PCR. A 485 bp fragment encoding a region just 5' of the kinase domain was generated from a full length cDNA clone (ATCC; 63207), using primers 5'-GCT GAT CCA GCA AAC ATT CA-3' and 5'-TGC CTT GCT TTT CAC TGT TG-3' and designated the NH_2_-terminal probe. A 1,166 bp fragment that corresponds to the COOH-terminal domain was generated by RT-PCR from total RNA isolated from FVB E14.5 embryos, using primers 5'-GCT CTA GAC AAT CCT GGA GGA GGA GAA GG-3' and 5'CTT CCT CGC TGC TGG TGG AAT TCT TGA GA-3' and designated the COOH-terminal probe. The cDNA fragments were separated on a 1% agarose gel and were gel purified using a gel extraction kit (Qiagen, Doncaster, VIC Australia) and cloned into the pGEM-T vector (Promega, Sydney NSW, Australia). Clones were sequenced and found to be 100% homologous with published FAK sequences.

In situ hybridization was performed on formalin-fixed paraffin sections (6 μm) using DIG-labeled riboprobes as described previously [36,44]. Briefly, sections were hybridized (55 °C) overnight with DIG-labeled sense or antisense transcripts (200 ng/ml) and washed with increasing stringency (6X-0.1X SSC) over a period of 2 h. Hybridization signal was visualized by incubation with anti-DIG antibody (1:1,000) conjugated to alkaline phosphatase (AP) and histochemical detection with NBT/BCIP (Roche Diagnostics, Castle Hill, NSW Australia), according to the manufacturer's instructions.

### Immunofluorescence

To localize FAK in ocular tissues, formalin-fixed paraffin embedded sections (6 μm) were dewaxed and rehydrated to water. Antigen retrieval was carried out by immersing sections in hot (60 °C) 0.01 M citrate buffer, heating for 5 min on high in a microwave, followed by 5 min at a lowered temperature setting to maintain the solution at or near boiling. Tissues were cooled to room temperature, equilibrated in phosphate buffered saline with 0.1% bovine serum albumin (PBS/BSA) for 10 min and blocked in PBS/BSA with 3% normal goat serum (Sigma-Aldrich, Castle Hill, NSW, Australia) for 30 min prior to incubation with the primary antibody (2-5 μg/ml). The primary antibodies used were a rabbit polyclonal antibody raised against a peptide at the NH_2_-terminus of FAK (SC557; Santa Cruz, Santa Cruz, CA), a rabbit polyclonal antibody raised against peptide at the COOH-terminus of FAK (SC558; Santa Cruz) and a mouse monoclonal antibody for phospho-Y^397^ FAK (MAB1144; Chemicon, Boronia, VIC, Australia). Reactivity was detected using AlexaFluor-488 conjugated anti-rabbit or anti-mouse secondary IgG (Molecular Probes/Invitrogen, Mt Waverley, VIC, Australia) diluted 1:500 in PBS/BSA at room temperature for 1-2 h. Following rinses in PBS/BSA, sections were stained with 1 μg/ml Hoechst dye (Sigma-Aldrich) to label nuclei, and mounted using DAKO fluorescent mounting medium (DAKO, Glostrup, Denmark). Control sections were incubated with nonimmune rabbit IgG or with the antibody pre-adsorbed with the corresponding peptide (SC558P or SC557P; Santa Cruz). Experiments were also carried out with antibody preadsorbed with a mixture of bovine α-, β-, and γ-crystallins (kindly provided by Prof Bob Augusteyn), to ensure the antibodies did not cross-react nonspecifically with the abundant crystallins. Results were visualized and captured using a Zeiss Axioskop-2 Plus microscope/Axiovision II software (Zeiss, Nth Ryde, NSW, Australia).

To localize FAK in cultured lens cells, epithelial explants were rinsed in PBS, fixed in 10% NBF or 4% paraformaldehyde with 0.2% Triton-X for 10 min, and washed in PBS. Samples were then blocked and incubated with antibody as described above. Nuclei were counterstained either with 1 μg/ml Hoechst dye or with 1 μg/ml propidium iodide for 5 min, washed in phosphate buffer, and then mounted for standard or confocal fluorescence microscopy as described above.

### Western blotting

Whole lenses from neonatal FVB and transgenic (OVE 591) or rat lens epithelial explants (untrimmed) were homogenized in extraction buffer (50 mM HEPES, 50 mM NaCl, 5 mM EDTA, 1% Triton-X, 50 mM sodium fluoride, 10 mM sodium phosphate, 10% protease, and phosphatase inhibitor cocktails [Sigma-Aldrich]). Following extraction, total protein of lens and explant extracts were quantified using the Bio-Rad Protein assay reagent (Bio-Rad, Regents Park, NSW, Australia). Equal amounts of protein for each sample were separated on 8-10% SDS-PAGE gels (Bio-Rad), under reducing conditions. Protein molecular weight marker (Kaleidoscope prestained standards, Bio-Rad) and positive control lysates from Jurkat cells (Chemicon) were included in each electrophoretic separation. Separated proteins were transferred to nitrocellulose membranes, blocked in 3% BSA in PBS with 0.1% Tween (PBST) for 1 h at room temperature and incubated with primary antibody (1 mg/ml in PBST) overnight at 4 °C. Membranes were washed in PBST and incubated for 1.5 h with the appropriate HRP-conjugated secondary anti-mouse or rabbit IgG (Bio-Rad), diluted 1:5000-1:10000. After washing with PBST, antibody binding was detected by enhanced chemiluminescence reagent (Amersham Biosciences/GE Healthcare, Rydalmere, NSW, Australia). To control for non-specific binding, immunoglobulin isotype control experiments were run in parallel.

To investigate the association of FAK with the cytoskeleton in epithelial and fiber cells, P9 rat lenses were dissected into epithelium (including lens capsule) and fiber preparations as described previously [36]. This involved tearing the posterior capsule at the posterior pole with fine forceps and removing the capsule, with epithelial cells attached, from the fiber mass. To ensure no contamination with adherent fibers or fiber fragments, the lens explants were trimmed to remove the posterior capsule and peripheral epithelial regions. The dissected tissues were rinsed in PBS and extracted in fresh RIPA buffer (0.1% SDS, 1% deoxycholate, 1% Triton X-100, 150 mM NaCl, 5 mM EDTA, 10 mM Tris pH7.4), containing a protease inhibitor cocktail (1 tablet/10 ml RIPA buffer; Roche Diagnostics) and additionally 1 mg/ml PMSF, leupeptin, aprotinin, and pepstatin A (Sigma-Aldrich). Extraction was performed using three rapid freeze-thaw cycles, using alternate liquid nitrogen and ice baths, and grinding using eppendorf pestles (Astral Scientific, Gymea NSW, Australia). The lysate was allowed to stand on ice for 30 min and then centrifuged at 800x g for 5 min. The supernatant was collected and centrifuged at 13,000x g at 4 °C for 30 min. The supernatants were collected and pellets were resuspended in fresh RIPA buffer. Protein concentrations were determined for all samples by the Micro BCA assay (Pierce, Paddington, QLD, Australia) and equal amounts of protein (6-10 μg) were separated on 10% SDS-PAGE gels under nonreducing or reducing conditions before transfer at 4 °C for 2 h onto Immobilon-PVDF membranes (Millipore, Billerica, MA) using a transblot apparatus (Bio-Rad). Blots were washed in Tris-buffered saline with 0.1% Tween-20 (TBST), blocked overnight in 3% BSA (Sigma-Aldrich) in TBST and incubated overnight with FAK antibodies (SC557, SC558) as described above. Antibody reactivity was detected by chemiluminescence as described above.

## Results

### FAK mRNA is expressed throughout lens development

**Figure 1:**
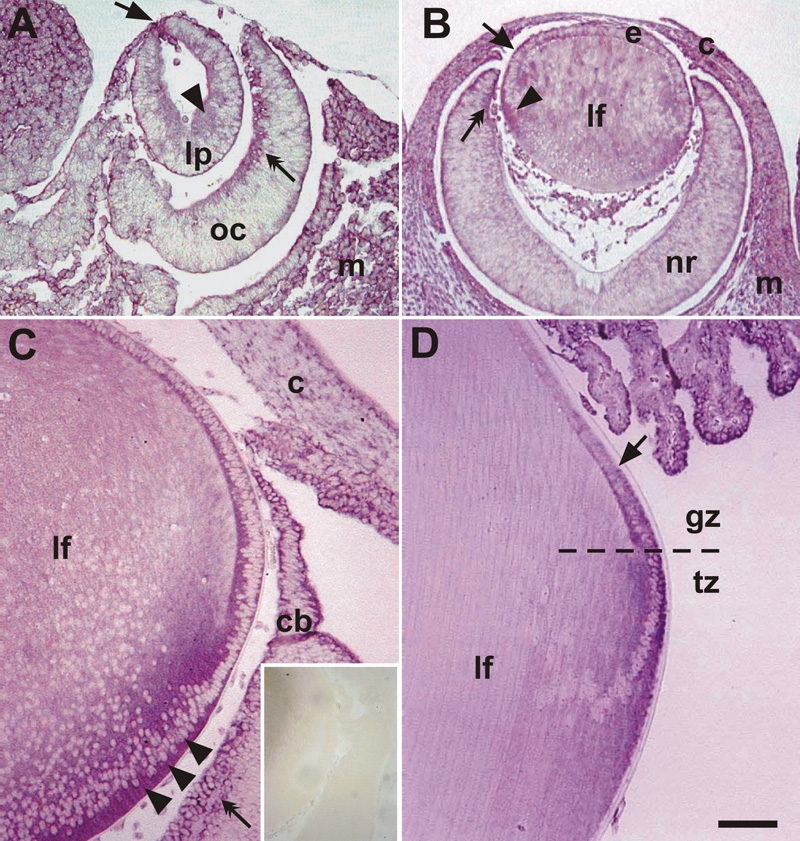
FAK mRNA expression during lens development. **A**: At E11.5, FAK was intensely expressed at the point of closure of the lens pit (arrow) with weaker expression detected at the apical cytoplasm of posterior cells of early pit/vesicle (arrowhead). Strong signals were also found along the inner optic cup (oc, double arrow) and in the extra-ocular mesenchyme (m). **B**: At E13.5, distinct FAK expression was detected in elongating fibers (arrowhead) of the late lens vesicle, while patchy expression (arrow) was detected in anterior epithelial cells (e). In the neural retina expression was predominantly found in the peripheral retina (double arrow) with little to no expression detected in the central regions. The developing cornea (c) and extra-ocular mesenchyme were also positive for FAK mRNA. **C**: At P1, FAK was expressed intensely in elongating fibers (lf), particularly in the basal cytoplasm (arrowheads). Weaker expression was detected in epithelial cells. Expression was also detected in the ciliary body (cb), corneal (c) epithelium, and ganglion cells of inner retina (double arrow). No staining was observed with the sense control probe (**C**, inset). **D**: At P21, strong FAK signal was detected in the lens transitional zone (tz) and posterior part (arrow) of the germinative zone (gz), whereas the more anterior epithelial cells and the mature fibers (lf) showed no expression. Strong expression was also detected in the ciliary body. Similar results were obtained with probes to 5' or 3' regions of the FAK cDNA. The scale bar in **A** represents 70 μm; in **B**, 150 μm; in **C** and **D**, 100 μm; and in **C**, inset, 300 μm.

To determine the spatio-temporal expression of FAK mRNA expression during lens morphogenesis, in situ hybridization was carried out using probes directed to the 5' and 3' regions of the FAK coding domain. Both probes produced similar patterns of expression. During early stages of lens morphogenesis, FAK was expressed ubiquitously in most ocular tissues, but was particularly strongly expressed in tissues undergoing morphogenetic movements and differentiation. For instance at E11.5, strong signal was detected in the apical lens pit, with distinct expression detected in the apical cytoplasm of the posterior pit cells (Figure 1A). FAK expression became more distinct in elongating fibers and anterior epithelium of differentiating lens vesicle at E13.5 (Figure 1B). At P1, FAK mRNA was detectable in the apical cytoplasm of both equatorial and anterior epithelial cells and was strongly localized in the basal cytoplasm of differentiating lens fibers (Figure 1C). Experiments with the sense probe failed to reveal any distinct hybridization signal at P1 (Figure 1C, inset) or at any age. At P21, FAK mRNA was not detectable in the anterior epithelial cells, but expression was initiated in cells in the posterior region of the lens germinative zone and became more strongly expressed in the transitional zone (Figure 1D).

Throughout ocular development, distinct FAK expression was also detected in the optic cup as it differentiates into neural retina. Particularly strong reactivity was associated with the inner optic cup at E11.5, the peripheral optic cup at E13.5, the ganglion cell layer at P1, and the ciliary body at P1 and P21 (Figure 1).

### Localization of FAK protein

**Figure 2:**
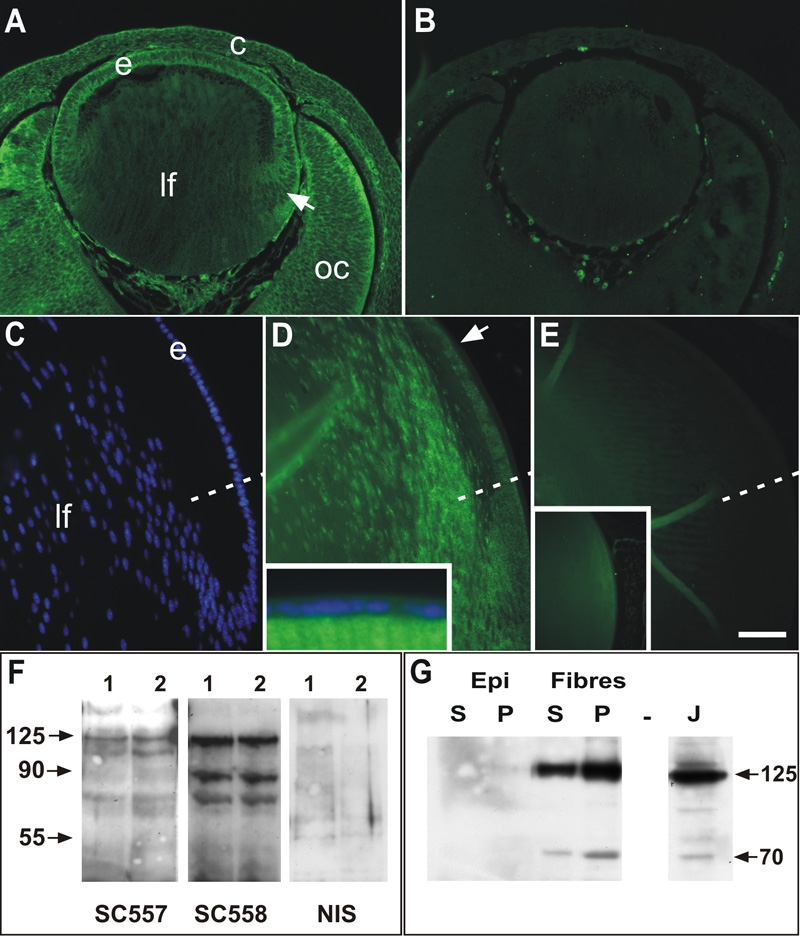
Immunolocalization of FAK in lens. Peptide antibodies to NH_2_-terminus (SC557) and COOH-terminus (SC558) domains of FAK were used to determine the presence of FAK protein in embryonic and postnatal lenses. **A**: Strong FAK reactivity in lens epithelium (e) and differentiating fibers (arrow) in E13.5 lens using the SC558 antibody. More mature fibers (lf) in the center of the lens showed little staining. Reactivity with this antibody was predominantly cytoplasmic and membrane-associated. Distinct staining was also detected in the optic cup (oc) and overlying cornea (c). **B**: SC558 pre-adsorbed with COOH-terminal peptide showed no reactivity in any ocular tissues; staining of blood cells is an autofluorescence artefact. **C**: Hoechst staining of section in **D**, showing epithelial (e) and fiber (lf) cells in the equatorial region of a P21 mouse lens. The equator is indicated by the dashed line in **C**-**D**. **D**: Distinct punctate reactivity for FAK (SC557 antibody) was detected in the epithelial cells of the posterior germinative zone (arrow) but not in anterior epithelial cells (inset). Reactivity was more intense in the transitional zone and in the cortical fibers and decreased in the more mature fiber cells. **E**: Non-immune serum controls showed no specific staining in sections from P21 lenses and preadsorption of SC557 with NH_2_-terminal peptide abolished almost all staining in the lens (inset). **F**: Western blots of whole lens extracts from two separate preparations probed with the SC557 and SC558 and non-immune rabbit IgG (NIS). SC557 revealed a distinct band at the predicted molecular mass of 125 kDa as well as lesser bands at about 110 kDa and about 65 kDa. SC558 also revealed the predicted 125 kDa as well as lower molecular weight species at about 85 kda and about 65 kDa. Membranes probed with the non-immune IgG showed no specific bands. **G**: Western blots of detergent-separated extracts (supernatant and pellet) from trimmed epithelial and fiber cell preparations (P9 rats), probed with the SC-558 antibody. FAK reactive bands at 125 and about 70 kDa were not detected in either soluble (S) or pellet (P) fractions from epithelial (Epi) cells, but were clearly detectable in both fiber cell preparations with more detectable in the cytoskeleton-associated fraction (P). J indicates Jurkat cell extract used as positive control. The scale bar in **A** and **B** represents 75 μm; in **C**-**E**, 50 μm; and in the insets, 125 μm.

Immunofluorescence experiments on E13.5 and P21 lenses showed similar patterns of FAK protein localization (Figure 2) to the mRNA expression patterns (Figure 1). In E13.5 lenses, there was distinct cytoplasmic and membrane labeling of the lens epithelium and elongating fibers with the SC558 antibody (Figure 2A). In the central differentiated fibers, very little FAK immunoreactivity was detected. Staining was also detected in the developing cornea and the optic neuroepithelium, particularly in the peripheral optic cup. Pre-adsorption of the antibody with the peptide used to raise the antibody completely blocked staining in all tissues (Figure 2B), whereas pre-adsorption with crystallins had no effect (data not shown). In P21 lenses, FAK reactivity (SC557 antibody) is detected in the posterior germinative zone of the lens and then gradually increases in the transitional zone and cortical regions where fiber cells undergo differentiation (Figure 2D). Reactivity was not detected in the anterior epithelium (Figure 2D, inset). Similar to the COOH-terminal antibody, the NH_2_-terminal antibody appeared to label cytoplasm and membranes, but was additionally detectable in fiber nuclei (Figure 2C,D). No reactivity was detected if the antibody was replaced with nonimmune IgG (Figure 2E) and preadsorption of the SC557 antibody with the peptide antigen greatly blocked reactivity (Figure 2D, inset). Pre-adsorption with crystallins had no effect (data not shown).

Western blots of neonatal whole lens extracts confirmed that both antibodies identified the predicted 125 kDa molecular weight band (Figure 2F,G), migrating in parallel with that found in the FAK positive control extracts from Jurkat cells. In addition, the NH_2_-terminal antibody showed a minor band at about 100 kDa and a diffuse band at about 60-75 kDa, whereas the COOH-terminal antibody revealed distinct bands at about 70 and 85 kDa. Incubation of the blots with control nonimmune IgG revealed no specific bands (Figure 2F).

To determine whether FAK was associated with the cytoskeleton in epithelial and fiber cells, P21 rat lenses were dissected into epithelial and fiber preparations and then extracted to prepare Triton-soluble (S) and -insoluble fractions (P). Probing with the COOH-terminal antibody revealed the presence of 125 kDa FAK in both fractions of fiber cells but was rarely detected in epithelial fractions (Figure 2G). Interestingly, the 70 kDa band detected with this antibody was predominantly detected in the P fraction and variably in the S fraction of fibers. Similar to the 125 kDa band, it was not detected in epithelial fractions (Figure 2G). A 43 kDa fragment was variably detected in both fractions.

### Localization of phosphorylated Y^397^-FAK

**Figure 3:**
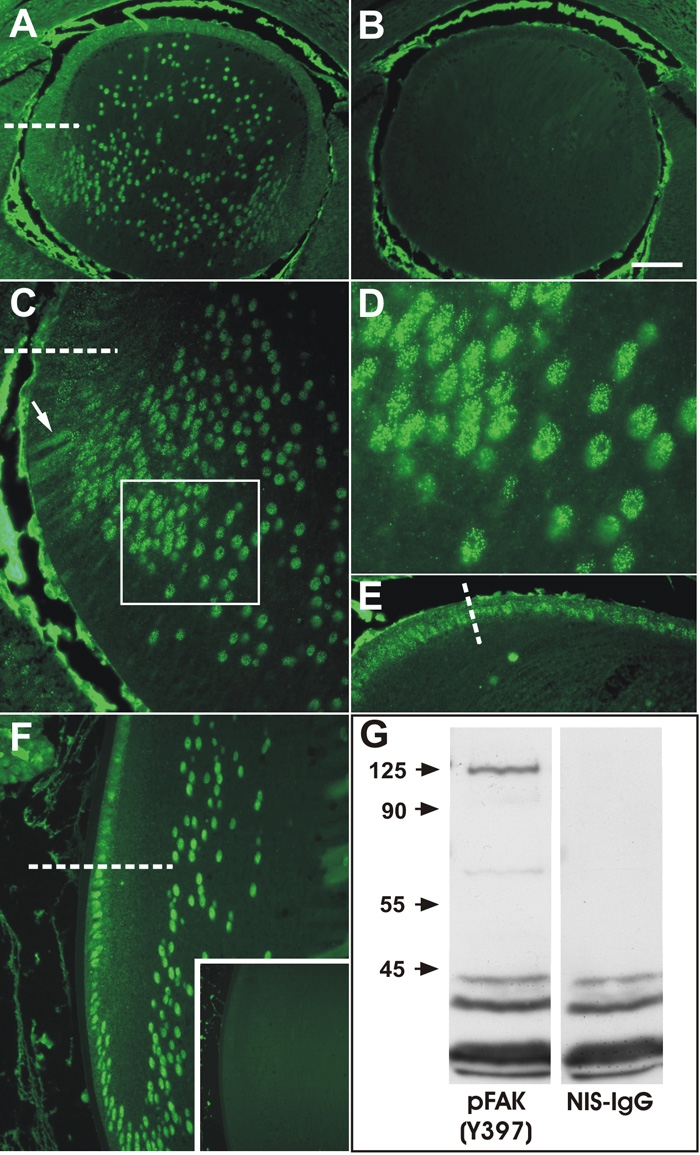
Immunolocalization of phosphoY^397^-FAK. **A**: At E14.5 phosphorylated-FAK was detected as punctate reactivity throughout the anterior epithelium, associated predominantly with membranes and in the cytoplasm. In the transitional zone below the equator (dashed line) and in fiber cells, reactivity became increasingly associated with the fiber cell nuclei. **B**: Incubation with non-immune mouse IgG revealed no specific staining in the lens; non-specific reactivity is present in the aqueous and vitreous chambers. **C**: At E17.5 a similar pattern of reactivity was detected, with reactivity being predominantly associated with membranes of epithelial cells and early differentiating fibers (arrow) but becoming increasing punctate nuclear as fibers elongate and differentiate into fibers (box). **D**: High magnification view of boxed region in **C**, showing the distinct nuclear punctate reactivity. **E**: High magnification view of epithelial cells anterior to the equator (dashed line) showing predominantly cytoplasmic and occasional nuclear punctate reactivity. **F**: A similar pattern of reactivity was present at P21, with epithelial cells in the posterior germinative zone showing predominantly cytoplasmic reactivity and cells below the equator showing predominantly nuclear reactivity. **G**: Western blots of whole lens extracts showed a predominant specific band at the predicted 125 kDa molecular mass and a faint minor band at about 65 kDa. Incubation with non-immune IgG showed no specific bands and non-specific staining of low molecular weight complexes (<45 kDa). The scale bar in **A**, **B**, and **F** indicates 100 μm; in the inset 250 μm; in **C**, and **F**, 40 μm; and in **D** 16 μm.

To investigate the activation of FAK during fiber differentiation we examined the distribution of FAK with a monoclonal antibody directed at the tyrosine-397 phosphorylated form of FAK (FAK-pY^397^). In embryonic mouse lenses at E14.5, distinct reactivity for FAK-pY^397^ was detected throughout the lens epithelium and in the transitional zone as a granular reactivity (Figure 3A), which was not detectable in sections incubated with nonimmune mouse IgG (Figure 3B). Similarly, in E17.5 lenses, reactivity in the epithelium was predominantly in the cytoplasm and occasionally associated with cell nuclei (Figure 3C-E). In the transitional zone of E17.5 lenses, reactivity appeared to be predominantly associated with cell membranes (Figure 3C,E). However, in differentiating fibers of these lenses, reactivity for FAK-pY^397^ became increasingly localized in the nuclei of cortical and central fibers (Figure 3A,C). At higher magnification, the reactivity was distinctly punctate within the nucleus but appeared to be excluded from the nucleolus (Figure 3D). A similar pattern was detected in P21 lenses, where the reactivity was predominantly cytoplasmic in the germinative zone and became increasingly nuclear in the transitional and cortical fibers (Figure 3F). Control sections incubated with non-immune IgG showed no staining (Figure 3F, inset). Western blots of whole lens extracts revealed predominantly the predicted 125 kDa band for FAK and a minor band migrating at about 65 kDa (Figure 3G).

### FAK Expression and phosphorylation is modulated by FGF2

**Figure 4:**
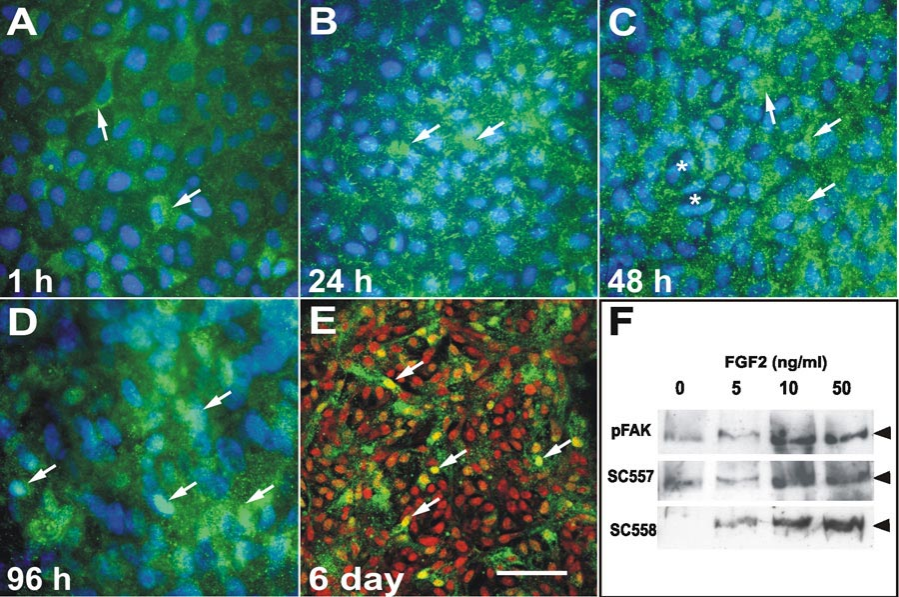
Effect of FGF2 on FAK expression and localization. Immunolocalization of phosphoY^397^-FAK (green) in untrimmed explants treated with FGF2 for 1 h (**A**), 24 h (**B**), 48 h (**C**), 96 h (**D**), and 6 days (**E**) and counter-stained with Hoechst (blue) or propidium iodide (red) to label nuclei. **A**: After 1 h of culture occasional epithelial cells (arrows) showed distinct punctate cytoplasmic reactivity for phospho-FAK. **B**: Reactivity increased after 24 h and almost all cells in the explant showed distinct punctate reactivity suggestive of adhesion junctions. **C**: A similar pattern of expression was observed at 48 h. Cells that were starting elongate (arrows) showed strong reactivity, whereas cells that had undergone division (asterisk) showed less distinct staining. **D**: By 96 h the elongating fiber cells showed distinct punctate FAK reactivity, some of which appeared to be present in the nucleus (arrows). As differentiating explants are multilayered we used confocal microscopy to confirm the nuclear localization. **E**: Confocal microscopy on explants cultured for 6 days in the presence of FGF2 showed distinct punctate phospho-FAK reactivity (green) in the cytoplasm of elongated fiber cells. Co-staining with propidium iodide, which labels nuclei red clearly showed nuclear staining (yellow) for phospho-FAK (arrows). **F**: Western blots of explants treated with FGF2 (0, 5, 10, or 50 ng/ml) for 5 days and probed with phosphoY^397^-FAK (pFAK), NH_2_-terminal (SC-557) and COOH-terminal (SC-558) antibodies. In control explants (0 ng/ml FGF2) low levels of FAK were detected. A slight increase in FAK expression and phosphorylation was detected with a proliferative dose of FGF2 (5 ng/ml). At doses of FGF2 that induce cell migration (10 ng/ml) and differentiation (50 ng/ml) there was marked increase FAK expression and phosphorylation. The scale bar in **A**-**D** indicate 15 μm and in **E**, 30 μm.

The developmental expression patterns of FAK imply that it is involved in processes of lens morphogenesis and differentiation. As the lens matures and these processes slow, FAK becomes restricted to cells exiting the cell cycle in the germinative zone, undergoing migration at the equator and differentiation in the transitional zone. Since FGFs are major regulators of cell proliferation, migration, and differentiation [10], we examined whether FAK expression is regulated by FGF. Exposure of lens epithelial explants to a fiber differentiating dose (100 ng/ml) of FGF2 induced a gradual increase in the expression of FAK-pY^397^ from 24 h to 6 days of culture. After 1 h of FGF2 exposure, only occasional cells showed significant reactivity for FAK-pY^397^ (Figure 4A), similar to control explants (not shown). However, after 24 h, there was a marked increase in the amount of reactivity for FAK-pY^397^ in explants (Figure 4B). Reactivity was at the membrane surface and appeared to be consistent with increased formation of focal adhesions. After 48 h of FGF2 exposure, cells became more elongated in shape and appeared to be more migratory. There is also evidence of mitosis. In these explants there was further increased staining for FAK-pY^397^ in most cells, except those that were obviously undergoing mitosis (Figure 4C). After 96 h of FGF2 treatment, the explants were considerably thickened as cells start to elongate and form fiber cells. In these explants, cells still showed the punctate, focal adhesion type staining, but there appeared to be increased staining of cell nuclei (Figure 4D). To ensure that the reactivity was indeed nuclear and not artifactual due to layering of the explant, confocal microscopy was used determine whether FAK-pY^397^ was present in the nucleus. Costaining of the explants for FAK-pY^397^ (green fluorescence), and with propidium iodide to label nuclei (red fluorescence), clearly showed that FAK-pY^397^ was present in the nuclei of many cells that were undergoing fiber differentiation in response to FGF2 (Figure 4E).

To investigate the dose-response characteristics of FAK expression with FGF2 exposure, explants were cultured for 5 days in the presence of 0, 5, 10, and 50 ng/ml of FGF2. Previous studies [45] have shown that 5, 10, and 50 ng/ml of FGF2 are half-maximal concentrations for lens cell proliferation, migration, and differentiation, respectively. Western blotting of explants treated with varying doses of FGF2 showed that there was very little change in the levels of FAK, or its phosphorylation, in explants treated with the proliferative concentration (5 ng/ml) of FGF2 for 5 days when compared to controls. However, treatment with concentrations that elicit migration and differentiation responses (10 and 50 ng/ml) showed dramatic changes in FAK expression and phosphorylation (Figure 4F).

### Altered FAK expression and activation in transgenic (OVE591) lenses

Previous transgenic studies have shown that TGFβ-like signals are required for terminal fiber differentiation. Moreover, cells expressing dominant-negative TGFβ receptors have defective migratory responses to FGF2 and abnormal actin filament assembly [12]. To investigate this further, we examined the expression and activation of FAK in lenses of transgenic mice (OVE591) that harbor a transgene expressing truncated TβRII in the lens fibers.

**Figure 5:**
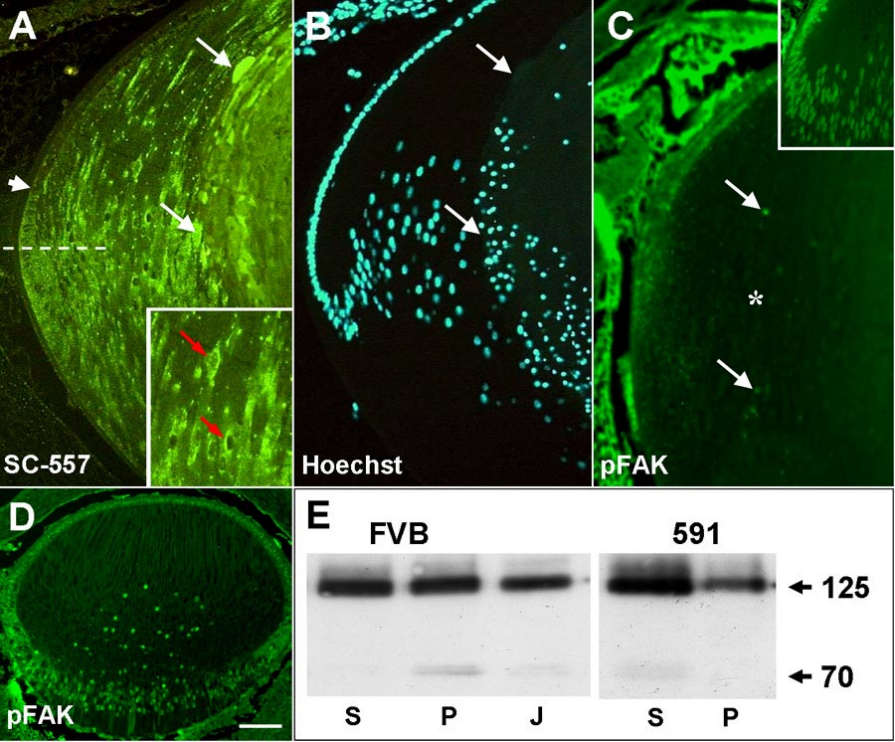
Expression of FAK in OVE591 transgenic lenses. **A**: Immunolocalization of FAK with the NH_2_-terminal antibody (SC557) in a P3 transgenic lens showing normal initiation of FAK expression in the posterior germinative zone (arrowhead) and expression in the transitional zone below the equator (dashed line). However, in more mature cortical and central fibers, FAK protein appears to aggregate abnormally in the cytoplasm and in perinuclear compartments (red arrows, inset) and is excluded from some nuclei. In the degenerate central lens fibers, intense reactivity for FAK is detectable in swollen fiber cells (arrows). **B**: Hoechst stained section shown in **A** showing where terminal differentiated fibers start to degenerate (arrows). **C**: Immunolocalization with the phosphoY^397^-FAK antibody on P2 lenses, just prior to appearance of the phenotype, showed greatly reduced expression of phosphorylated FAK in the fiber mass (asterisk) compared to wild-type lenses (inset). Some nuclear FAK is detected in nuclei of a few cortical fibers (arrows). **D**: In embryonic (E17.5) lenses, 7 days prior to phenotype initiation, expression of phospho-FAK is still present in the epithelium and fibers, similar to wild-type. **E**: Western blots of detergent-separated whole lens extracts (supernatant and pellet) from P3 FVB and OVE591 lenses, probed with SC558. In wild-type FVB lenses, equivalent amounts of 125 kDa FAK are present in the soluble (S) and in the cytoskeleton-associated (P) fractions, whereas in OVE591 lenses there was a shift of FAK from the cytoskeletal (P) to the soluble (S) fraction. A FAK-reactive band of approximately 70 kDa was evident in the P but not the S fraction of wild-type FVB lenses. In the 591 mutant lenses this band was absent from the P fraction but weakly detected in the S fraction. J indicates the Jurkat cell extract positive control. The scale bar in **A**-**D** indicate 100 μm; in **A**, inset, 200 μm; and in **C**, inset; 150 μm.

Immunolocalization of FAK with the NH_2_-terminal antibody in P21 lenses showed there was normal initiation of FAK expression in the posterior germinative zone and high levels of expression in the cortical fibers (Figure 5A). Similar to wild-type lenses (Figure 2B), the reactivity was punctate and localized to the cytoplasm. However, the FAK reactivity appeared to persist in the cortical differentiating fibers of transgenic lenses and became increasingly localized to a perinuclear compartment (Figure 5A, inset). In the more cortical fibers that have started to swell and undergo degeneration there appeared to be greatly increased reactivity for FAK (compare Figure 2D and Figure 5A). Localization of FAK-pY^397^ reactivity in neonatal lenses, at the time the phenotype first becomes apparent (P2), revealed some punctate cytoplasmic staining for FAK-pY^397^ in epithelial cells and in transitional zone fibers. However, staining was greatly reduced or absent in the cortical fibers, both at the membrane and in the nuclei (Figure 5C) when compared to wild-type lenses (Figure 5C, inset). By contrast, FAK-pY^397^ was still detectable in E17.5 transgenic lenses, before the lens phenotype becomes apparent (Figure 5D). Western blots of Triton-soluble (S) and -insoluble fractions (P) from wild-type and transgenic lenses, showed a dramatic reduction of 125 kDa FAK associated with the cytoskeleton in the insoluble (P) fraction, and increased reactivity in the soluble (S) fraction (Figure 5E). Interestingly, the 70 kDa fragment, which is normally found predominantly in the P fraction of FVB mice, was absent from the P fraction in OVE5921 mice and weakly detected in the S fraction (Figure 5E). A 43 kDa fragment was variably detected in both fractions (not shown).

## Discussion

Consistent with the finding that inhibition of integrin signaling disrupts early stages of lens morphogenesis [38], the expression data obtained in this study, using both mRNA and protein probes, indicate that FAK plays a role during morphogenesis and differentiation of the lens. FAK is distinctly localized in the invaginating lens pit and optic cup as well as in the proliferating anterior epithelial cells and in differentiating fibers during the rapid phase of lens growth (E14.5-P1). However, as the lens matures postnatally, FAK expression becomes more restricted. In the lens epithelium of weanling mice, FAK expression is absent from the relatively quiescent anterior cells, but is initiated in the posterior germinative zone, where epithelial cells exit the cell cycle, and is maintained in the transitional zone, where cells commence elongation and fiber differentiation. Thus FAK is expressed in regions of the lens where cells exit the cell cycle, migrate, and differentiate.

An antibody directed to the major phosphorylated form of FAK (pY^397^-FAK) revealed similar patterns of FAK activity, with active FAK present throughout the embryonic lens. As the lens matures, it becomes more restricted to the equatorial region, where cells exit the cell cycle, migrate, and differentiate. Consistent with this spatial pattern, FGF stimulated increased FAK expression and phosphorylation at concentrations that induce cells to migrate and differentiate but not at concentrations that stimulate cell proliferation. The pattern of pY^397^-FAK expression in the lens is very similar to the pattern of phosphorylated Src [46]. Src can be recruited into a complex with FAK once FAK is phosphorylated on Y^397^ and subsequently Src mediates phosphorylation of FAK at other tyrosine residues, thus ensuring complete activation of FAK and permitting binding of other proteins and kinases [40]. In lens cells, Src activity has been shown to regulate the transition from proliferation and differentiation [47], as well as Na, K-ATPase activity [48] and induction of cataracts [49]. While Src and FAK are coordinately regulated during differentiation of pigmented epithelial cells [50], it is not known how FAK and Src modulate each other in the lens.

While all three antibodies showed localization of FAK in the cytoplasm and in association with cell membranes, the NH_2_-terminal and pY^397^-FAK antibodies also revealed punctate nuclear reactivity in differentiating fibers. This was particularly evident with the pY^397^-FAK antibody. Moreover, in vitro experiments indicate that epithelial cells induced to differentiate with FGF2 also accumulated pY^397^-FAK reactivity in the nuclei, suggesting that this phenomenon is regulated by FGF. The fact that this accumulation did not occur in all cells of FGF-treated explants indicates that other factors may also be involved.

The presence of NH_2_-terminal FAK fragments in the nucleus has been detected in other cell types undergoing apoptosis and it has been proposed that they have signaling functions during apoptosis and cell adhesion [51-54]. Cleavage of FAK, to yield both COOH-terminal and NH_2_-terminal fragments in apoptotic cells, has been shown to be mediated by caspase-3, giving rise to various fragments from 55-90 kDa, depending on the species [55]. The presence of similar fragment sizes (60-100 kDa) in lens extracts as well as nuclear aggregates of FAK reactivity in cortical lens fibers, coincident with regions of caspase-3 expression [6,9,56], suggest that there is similar cleavage of FAK by caspase-3 and accumulation of NH_2_-terminal FAK fragments in the lens fiber nucleus during differentiation.

While cleavage of FAK is thought to be a mechanism to regulate its activity in adhesion junctions [40], the role of FAK in the nucleus is still unclear. Jones et al. [51] showed that transfection of glioblastoma cells with an NH_2_-terminal FAK fragment increased nuclear FAK accumulation but did not induce increased apoptosis and concluded that NH_2_-terminal FAK is not, by itself, proapoptotic and that nuclear accumulation is a response to an apoptotic stimulus. By contrast, overexpression of a COOH-terminal FAK fragment does induce apoptosis, presumably by competing with intact FAK in adhesion junctions and by attenuating EGFR signaling. In the lens, FAK expression, activation, and accumulation of nuclear FAK aggregates is induced by FGF, suggesting that FAK may act as the integration point between growth factor and ECM signals during fiber differentiation. In normal lens development, terminal fiber cell differentiation involves loss of nuclei and organelles by a modified apoptotic mechanism, whereby the cell cytoplasm and membranes remain intact [7]. As FAK is known to mediate survival signals, it is plausible that the balance of full-length FAK and COOH-terminal fragments in adhesion complexes plays a role in regulating apoptosis to ensure denucleation without cytoplasmic blebbing of fibers. However, such a role for FAK in fibers will need to be investigated further by mutational strategies, designed to alter the ratio of FAK and COOH-terminal fragments during fiber differentiation.

Terminal fiber differentiation requires signaling by TGFβ family members. Inhibition of BMP signals by extracellular antagonists or by dominant negative receptors inhibits lens fiber cell elongation [14,15]. Ectopic expression of a dominant-negative TGFβ receptor has been shown to decrease Smad2 phosphorylation and to disrupt fiber differentiation in transgenic mice (OVE591) [11,12]. However, recent studies of mice with conditional inactivation of TβRII in the lens showed no phenotype and it was suggested that overexpression of dominant-negative TβRII receptors resulted in nonspecific effects on lens differentiation [13]. Interestingly, the lens fibers of the conditional null mice showed no reduction in phospho-Smad2 reactivity, suggesting that loss of TβRII receptors is insufficient to inhibit Smad2 activation and that there may be some redundancy of function with other receptors. The lens expresses BMP, TGFβ, and activin receptors [11,44,57,58]. Taken together with evidence that TβRII can form functional signaling complexes with Alk1, Alk2, and Alk5 [59-61], this suggests that the dominant-negative receptor may inhibit several TGFβ-family pathways in the lens that converge on several smads, including Smad2.

The lens phenotype in TβRII dominant-negative transgenic mice (OVE591) appears at postnatal day 3 (P3) and analyses of epithelial cells in culture indicate that as these cells differentiate under the influence of FGF2, they fail to migrate and do not assemble actin stress filaments [44]. Analysis of FAK expression in these lenses shows that while FAK is normally expressed and activated at embryonic stages, it becomes disrupted from about postnatal day 2 (P2). FAK protein appears to be incorrectly targeted as it remains in perinuclear compartments of fiber cells and is not found at the membranes in mutant lenses. Consistent with this, there is decreased cytoskeletal association of FAK and dramatic loss of FAK phosphorylation in these lenses at P2, suggesting that disruption of FAK targeting and activation contributes to this phenotype. Interestingly, the 70 kDa FAK COOH-terminal reactive fragment is lost from detergent-insoluble fractions and appears in the detergent-soluble fractions, suggesting that this fragment also no longer associates with the cytoskeleton. While the identity and function of this fragment is still not clear, it is unlikely to represent FRNK as this migrates at about 45 kDa.

TGFβ is known to stimulate FAK activation in other systems, such as myofibroblast differentiation from fibroblasts [43,62,63] and in cancer cells [64-66], thus this change in FAK activation may occur as a direct consequence of loss of TGFβ family signaling in the lenses of these transgenic mice. However, the actin cytoskeleton is also essential for phoshorylation of FAK, as disruption of the actin cytoskeleton with cytochalasin-D abolishes FAK phosphorylation [40,67,68], thus the loss of FAK phosphorylation in these mutant lenses may be due to a primary disruption of the actin cytoskeleton. Interestingly, disruption of the actin cytoskeleton in cultured lenses with cytochalasin D results in a cortical cataract and cellular disruption [69,70] that bears some similarities to the cataract seen in OVE591 mice. The fact that loss of FAK phosphorylation is detectable on postnatal day 2, which is prior to the onset of the phenotype, suggests that this is an early event in the disrupted fiber differentiation process in these transgenic lenses and that the absence of TGFβ-like signals results in abnormal phosphorylation of FAK and this leads to abnormal actin cytoskeleton dynamics.

In summary, these studies show that FAK is expressed and activated during lens morphogenesis and differentiation. Its expression and activation during initiation of fiber differentiation at the lens equator appear to be modulated by FGF. Terminal differentiation of fibers also involves nuclear targeting of a phosphorylated NH_2_-terminal FAK fragment, which may play a role during fiber cell differentiation and denucleation. Moreover, during abnormal terminal fiber differentiation, in lenses expressing a dominant-negative TGFβ receptor, there is abnormal subcellular targeting, loss of cytoskeletal association, and decreased activation of FAK. These data suggest that integrin signaling via FAK plays important roles during initiation of lens differentiation by FGF and completion of terminal differentiation mediated by TGFβ family members.
